# Draft Genome Sequence of *Phaeobacter leonis* Type Strain 306, an Alphaproteobacterium Isolated from Mediterranean Sea Sediments

**DOI:** 10.1128/genomeA.00312-17

**Published:** 2017-06-29

**Authors:** Frédéric Gaboyer, Loïs Maignien, Mohamed Jebbar, Karine Alain

**Affiliations:** a Centre de Biophysique Moléculaire (CBM), Centre National de la Recherche Scientifique (CNRS), Orléans, France; b CNRS, Institut Universitaire Européen de la Mer (IUEM) – UMR 6197, Laboratoire de Microbiologie des Environnements Extrêmes (LM2E), Plouzané, France; c Université de Bretagne Occidentale (UBO, UBL), IUEM – UMR 6197, Laboratoire de Microbiologie des Environnements Extrêmes (LM2E), Plouzané, France; d Ifremer, UMR 6197, LM2E, Technopôle Pointe du Diable, Plouzané, France

## Abstract

*Phaeobacter leonis* strain 306^T^ is an alphaproteobacterium isolated from Mediterranean Sea sediments. It belongs to the genus *Phaeobacter*, which was recently proposed and is still poorly characterized. In an effort to better understand the fundamental aspects of the microbiology of this genus, we present here the 4.82-Mb draft genome sequence of *Phaeobacter leonis* strain 306^T^.

## GENOME ANNOUNCEMENT

The genus *Phaeobacter*, in the subclass *Alphaproteobacteria*, was proposed in 2006 and comprises strains previously classified in the genus *Roseobacter* (1). To date, seven *Phaeobacter* species have been formally described and are mostly represented by marine aerobic heterotrophic bacteria. These isolates are widely distributed in the marine environment and were isolated from the Arctic Ocean, the Mediterranean Sea, or the Yellow Sea. The presence of *Phaeobacter* species in oligotrophic and cold marine habitats may suggest that representatives of this genus have physiological characteristics in accordance with these environmental conditions. So far, only one study of comparative genomics has focused on the genus *Phaeobacter*. It revealed the existence of microdiversity and the presence of ecotypes within the species *Phaeobacter gallaeciensis* associated with differential ecological features (2). However, the few number of isolated and described species of *Phaeobacter* does not allow speculation about the evolutionary history of this group. Otherwise, some *Phaeobacter* species are also of pharmaceutical interest. This is notably the case of *Phaeobacter inhibens*, which synthetizes the tropodithietic acid (TDA) antibiotic.

In 2008, *Phaeobacter leonis* strain 306^T^ was isolated from Mediterranean Sea sediments. It was then formally described as an aerobic heterotroph representing the first species of *Phaeobacter* isolated from marine sediments (3). In order to better characterize the functional features of this recently recognized genus, the genome of *Phaeobacter leonis* strain 306^T^ has been sequenced.

The genome sequencing of *P. leonis* strain 306^T^ was performed using the Illumina MiSeq technology, with 2 × 250-bp read size, by the Fasteris Company (Plan-les-Ouates, Switzerland). Reads were assembled with Velvet (4) using the GenOuest Galaxy platform (5), with a 34-bp k-mer. The final assembly comprises 135 contigs >1,000 bp in size and provides 428× coverage of the genome. The longest contig has a size of 192,246 bp, and the *N*_50_ value is 62,560 bp. Coding sequences (CDSs) were detected with Glimmer 3.0 and annotated with the RAST service (6) and the MicroScope Microbial Genome Annotation and Analysis Platform (MaGe) (7). Transfer RNAs were analyzed with tRNAscan-SE (8).

The estimated genome size totals 4,823,053 bp, with a G+C content of 58.7%. It contains 5,578 putative genes, 3 *rrn* genes, and 43 tRNAs, with a coding density of 0.84. Among these genes, 78.4% and 52% could be classified, respectively, in Clusters of Orthologous Groups (COG) and in the SEED subsystems, and 25.4% were annotated as hypothetical proteins. No genes for TDA biosynthesis were predicted. Genes for flagellar assembly are present. Genes for pilus assembly, type II and type IV secretion systems, and 20 methyl-accepting chemotaxis proteins indicate that chemotaxis and attached lifestyles could be adaptive features in this species, although no genes for quorum sensing were predicted. Several copies of phage major capsid proteins, phage portal proteins, and phage terminases demonstrating prophage sequences were found in the chromosome, but no clustered regularly interspaced short palindromic repeat (CRISPR) region was found. Its closest relative is *Phaeoabcter arcticus*, with which it shares 98.7% 16S rRNA identity and 3,021 putative orthologs (54% of its genes).

### Accession number(s).

This whole-genome shotgun project has been deposited at DDBJ/ENA/GenBank under the accession no. MWVJ00000000. The version described in this paper is version MWVJ01000000.
